# A preliminary investigation assessing the viability of classifying hand postures in seniors

**DOI:** 10.1186/1475-925X-10-79

**Published:** 2011-09-09

**Authors:** Mojgan Tavakolan, Zhen Gang Xiao, Carlo Menon

**Affiliations:** 1 MENRVA Group, School of Engineering Science, Faculty of Applied Science, Simon Fraser University, 8888 University Drive, Burnaby, BC, V5A 1S6, Canada

## Abstract

**Background:**

Fear of frailty is a main concern for seniors. Surface electromyography (sEMG) controlled assistive devices for the upper extremities could potentially be used to augment seniors' force while training their muscles and reduce their fear of frailty. In fact, these devices could both improve self confidence and facilitate independent leaving in domestic environments. The successful implementation of sEMG controlled devices for the elderly strongly relies on the capability of properly determining seniors' actions from their sEMG signals. In this research we investigated the viability of classifying hand postures in seniors from sEMG signals of their forearm muscles.

**Methods:**

Nineteen volunteers, including seniors (70 years old in average) and young people (27 years old in average), participated in this study and sEMG signals from four of their forearm muscles (i.e. Extensor Digitorum, Palmaris Longus, Flexor Carpi Ulnaris and Extensor Carpi Radialis) were recorded. The feature vectors were built by extracting features from each channel of sEMG including autoregressive (AR) model coefficients, waveform length and root mean square (RMS). Multi-class support vector machines (SVM) was used as a classifier to distinguish between fifteen different essential hand gestures including finger pinching.

**Results:**

Classification of hand gestures both in the pronation and supination positions of the arm was possible. Classified hand gestures were: rest, ulnar deviation, radial deviation, grasp and four different finger pinching configurations. The obtained average classification accuracy was 90.6% for the seniors and 97.6% for the young volunteers.

**Conclusions:**

The obtained results proved that the pattern recognition of sEMG signals in seniors is feasible for both pronation and supination positions of the arm and the use of only four EMG channel is sufficient. The outcome of this study therefore validates the hypothesis that, although there are significant neurological and physical changes occurring in humans while ageing, sEMG controlled hand assistive devices could potentially be used by the older people.

## Background

Improving independent living of seniors and maintenance of their autonomy are compelling research goals for our society. Some simple activities of daily living such as opening and closing the screw cap of a bottle or turning a tap handle can be difficult tasks for a senior. By increasing the age, the skeletal muscles lose their strength [1]. In order to do everyday simple operations, seniors would need using assistive devices that could provide an additional force for their hand movements and also train their muscles [2].

A compelling challenge in the development of assistive devices is how to acquire information from input signals that provide us with the information regarding the action the user is undertaking. Acquiring the input signals from the neurological activity of the user would provide us with the desired information. sEMG is a suitable technique for evaluating and measuring the electrical activity produced by skeletal muscles and can also provide us with important information regarding neuromuscular disorders [3]. Using sEMG, we are able to detect the electrical signals generated by muscle cells when they are neurologically or electrically activated and if we interpret this information correctly, it can guide us towards the intention of the user [2,3].

EMG signals have been considered to control prosthetic hands and assistive devices. Different prosthetic hands have been prototyped including the Smart Hand [4] and the Cyber Hand [5]. Some EMG driven prostheses have also been commercialised; examples are the Otto Bock's Sensor Hand Speed [6] and the iLimb [7]. In the mentioned researches, the goal was to obtain a prosthetic hand that could perform movements similar to a human hand. A challenging part in the development of these prosthetic hands is the design of an intuitive control achieved by detection and interpretation of the user's neurological activity [8,9]. Whether used for controlling prosthetic, rehabilitative or assistive devices, sEMG signals should be processed to identify the intention of the user.

One of the main challenges related to the processing and classification of sEMG is related to the synergistic use of upper extremity muscles. For example, raising the shoulder to lift the forearm results in forearm signal changes [9]; similarly, contracting the index finger results in co-contraction of forearm muscles [10-12].

Different pattern recognition techniques have been used for classification of sEMG [2,3] and identification of hand gestures in young volunteers [13,14]. For example, multilayer perceptron [15,16], SVM [9,17-20], hidden markov model [21], neural networks [22], bayesian classifier [23] and fuzzy classifier [24-26] techniques have been proposed. Multiple features have been investigated including AR model coefficients [22,24,26,27], mean absolute value [27,28], slope sign changes [29,30], zero crossings [27-29], waveform length [29,30] and wavelet packet transform [15,31].

Most of the research has been performed with populations involving young healthy volunteers and amputees. Little research has however been carried out to assess if aging prevents a successful sEMG classification, which is needed to control assisted devices developed to augment force and reduce fear of frailty in the older people. It should be noted that there are significant neurological and physical changes occurring in humans while ageing [32]. This study therefore focuses on assessing the viability of classifying hand postures in seniors.

## Methods

### Data collection

**Figure 1:**
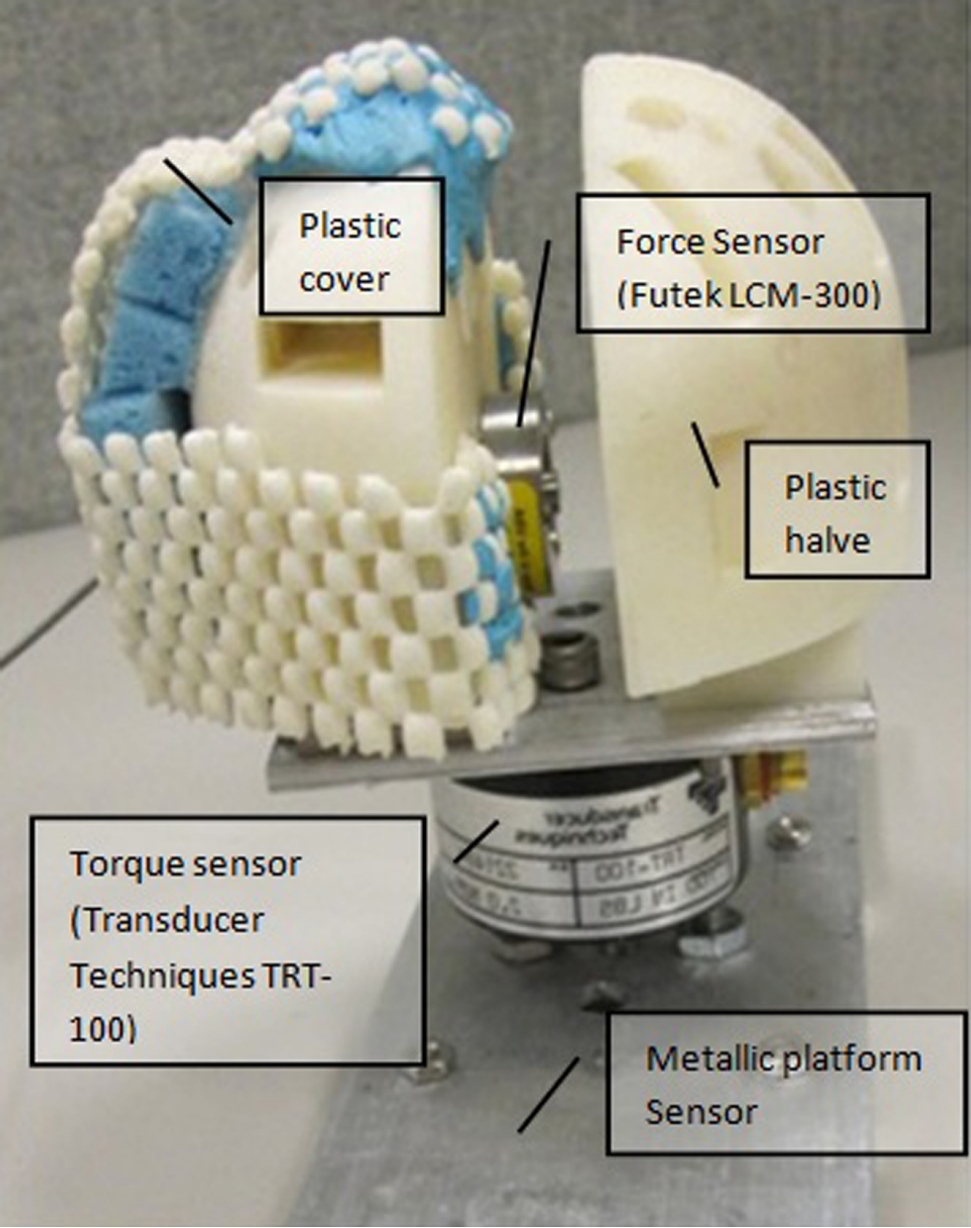
**Custom rig**.

A custom rig was used to measure hand force and torque exerted by the volunteers. The rig (see Figure 1) consisted of a force sensor (Futek LCM-300) which measured contraction force. This sensor was placed between two plastic halves, which formed together a semi-sphere to enable the volunteers to comfortably hold the rig with their hand. These two plastic halves were connected to a metallic platform through a torque sensor (Transducer Techniques TRT-100) that recorded torque produced by the volunteer while performing ulnar or radial deviation movements.

**Figure 2:**
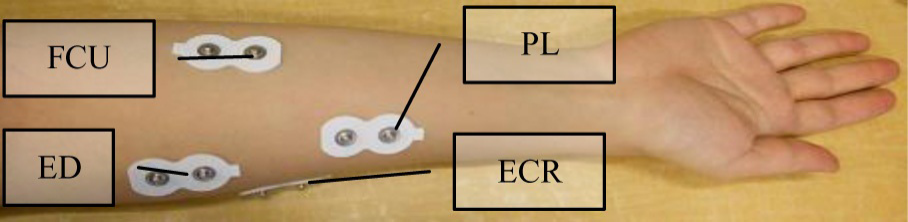
**Location of surface electrodes on the forearm**.

Guidelines presented in the sEMG for the non-invasive assessment of muscles (SENIAM) project [33] were followed to obtain a fine skin contact with the electrodes. According to these guidelines, the skin was cleaned with an alcohol swab and electrodes were placed at the locations shown in Figure 2. sEMG electrodes were attached to the volunteers' forearms using medical adhesive bands that made the electrodes' active faces adhere the skin.

sEMG signals were recorded from the following four muscles in order to detect movement of wrist and fingers [34]: Extensor Digitorum (ED), Palmaris Longus (PL), Flexor Carpi Ulnaris (FCU) and Extensor Carpi Radialis(ECR). Function of each muscle is summarized in Table 1. sEMG signals were acquired through a Noraxon system (Myosystem 1400L). A data acquisition board from National Instruments (USB-6289) was used in this study for acquiring both the sEMG signals and the data obtained from the custom rig used to measure hand force and torque. Since the EMG signal has usable energy in the 0-500 Hz range [35], the acquired sEMG signal was digitized at 1024 samples per second and stored on a computer through an application developed in LAbVIEW software. The developed LabVIEW application also had a graphical interface to enable volunteers visualizing force they were exerting during the tests. For each participant, the maximum force exerted to the rig was used to define the participant's maximum voluntary contraction (MVC). According to [36], the applied force should not exceed 40-50% of the MVC in order to prevent upper extremity musculoskeletal injuries. For this reason, all the protocols were defined to prevent exceeding this limit.

**Table 1:** Muscle function

Muscle	Function
FCU	Assists in wrist flexion with ulnar deviation

PL	Assists in wrist flexion

ED	Assists in extension of four fingers and the wrist

ECR	Assists in extension and radial abduction of the wrist

### Protocol

**Figure 3:**
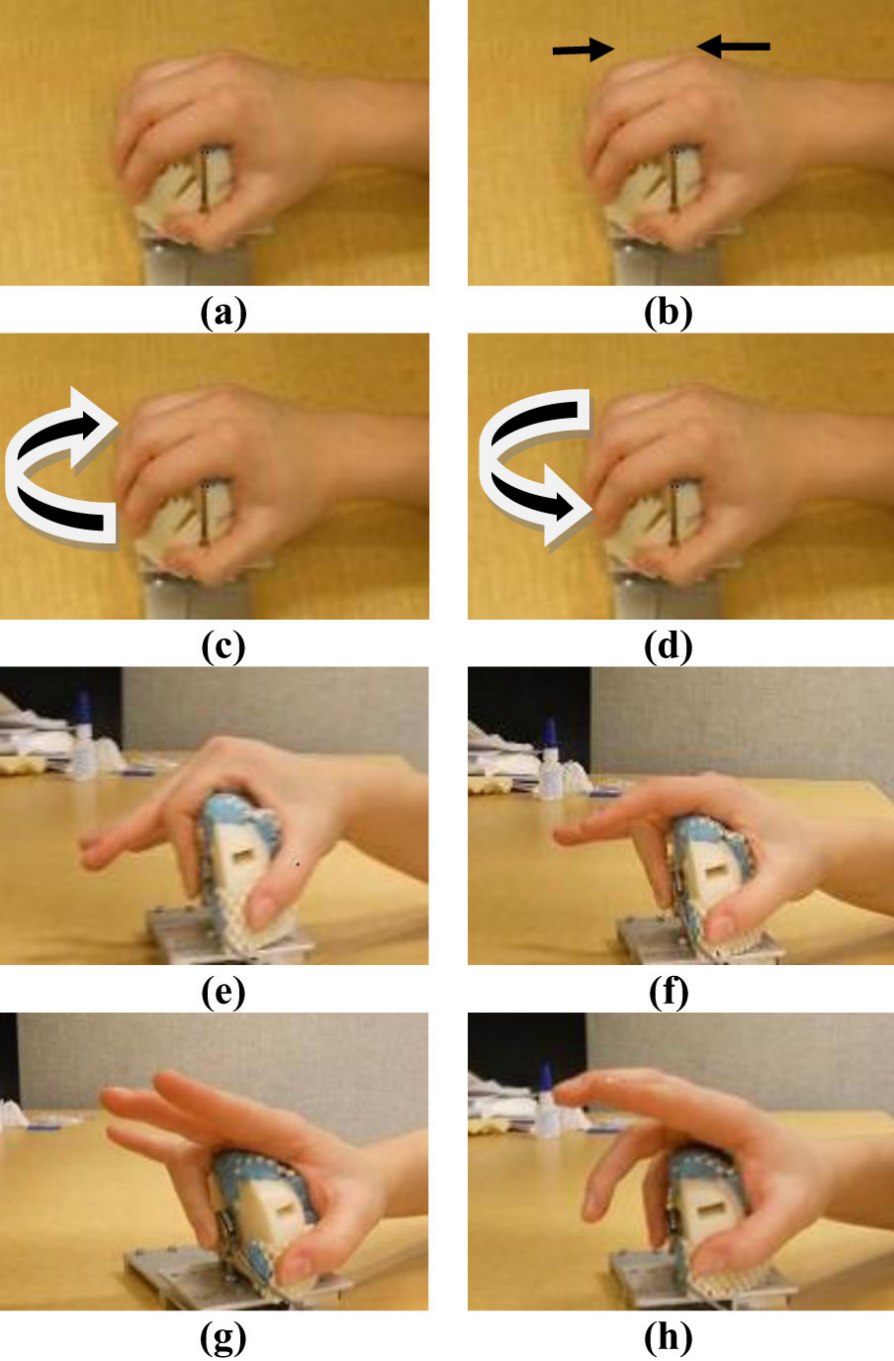
**Hand gestures and motions chosen for classification in the pronation position of the arm**. (a) rest, (b)grasp, (c) ulnar deviation, (d) radial deviation, (e)finger pinching: index finger, (f) finger pinching: middle finger, (g) finger pinching: ring finger, (h) finger pinching: little finger.

12 seniors (70 years old on average) and 7 young volunteers (27 years old on average) participated in this study. The Office of Research Ethics, Simon Fraser University approved this study and each senior signed a consent form. Each volunteer followed the eight predefined protocols summarized in Table 2. These protocols were defined to simulate simple activities of daily living involving the wrist and fingers such as opening and closing the screw cap of a jar or grasping an object. The identified protocols considered a combination of several hand movements including grasping, finger pinching, wrist ulnar/radial deviation and forearm pronation/supination. Each volunteer started at rest position as shown in Figure 3-a.

**Table 2:** Protocols

Protocols	Definitions	Arm position
Protocol A	Apply maximum force by squeezing the custom rig two times.	Pronation

Protocol B	Apply maximum torque for radial deviation two times and then apply maximum torque for ulnar deviation two times.	Pronation

Protocol C	Apply 50% MVC force while squeezing for three seconds. Repeat for three times.	Pronation

Protocol D	Apply 50% MVC torque for alternate radial and ulnar deviation for three seconds. Repeat for three times.	Pronation

Protocol E	Pinch two times with a comfortable force using thumb and index finger, then two times using thumb and middle finger, then two times using thumb and ring finger and finally two times using thumb and little finger.	Pronation

Protocol FC	Apply 50% MVC force while squeezing for three seconds. Repeat for three times.	Supination

Protocol FD	Apply 50% MVC torque for alternate radial and ulnar deviation for three seconds. Repeat for three times.	Supination

Protocol FE	Pinch two times with a comfortable force using thumb and index finger, then two times using thumb and middle finger, then two times using thumb and ring finger and finally two times using thumb and little finger.	Supination

In protocol A, as shown in Figure 3-b, the volunteer was asked to squeeze the custom rig with maximum force in pronation position of the arm for two times. The recorded maximum force was used to define MVC for squeezing.

In protocol B, as shown in Figures 3c-d, the volunteer was asked to apply maximum torque in ulnar and radial deviation for two times (pronation position of the arm). Maximum torques for ulnar and radial deviations were used to identify ulnar/radial MVCs.

In protocol C, the volunteer was asked to squeeze the custom rig at 50% of her/his MVC for 5 seconds (pronation position of the arm). The volunteer repeated this protocol three times. Using the graphical interface of the developed LabVIEW application, the volunteer had visual feedback for the force applied to the custom rig.

In protocol D, the volunteer was asked to alternate radial and ulnar deviation for 5 seconds at 50% of MVC (pronation position of the arm). The volunteer repeated this procedure three times.

In protocol E, as shown in Figures 3e-h, the volunteer pinched the force sensor firstly with thumb and index finger, secondly with thumb and middle finger, thirdly with thumb and ring finger, and finally with thumb and little finger (pronation position of the arm). The pinching was repeated two times for each combination of fingers.

**Figure 4:**
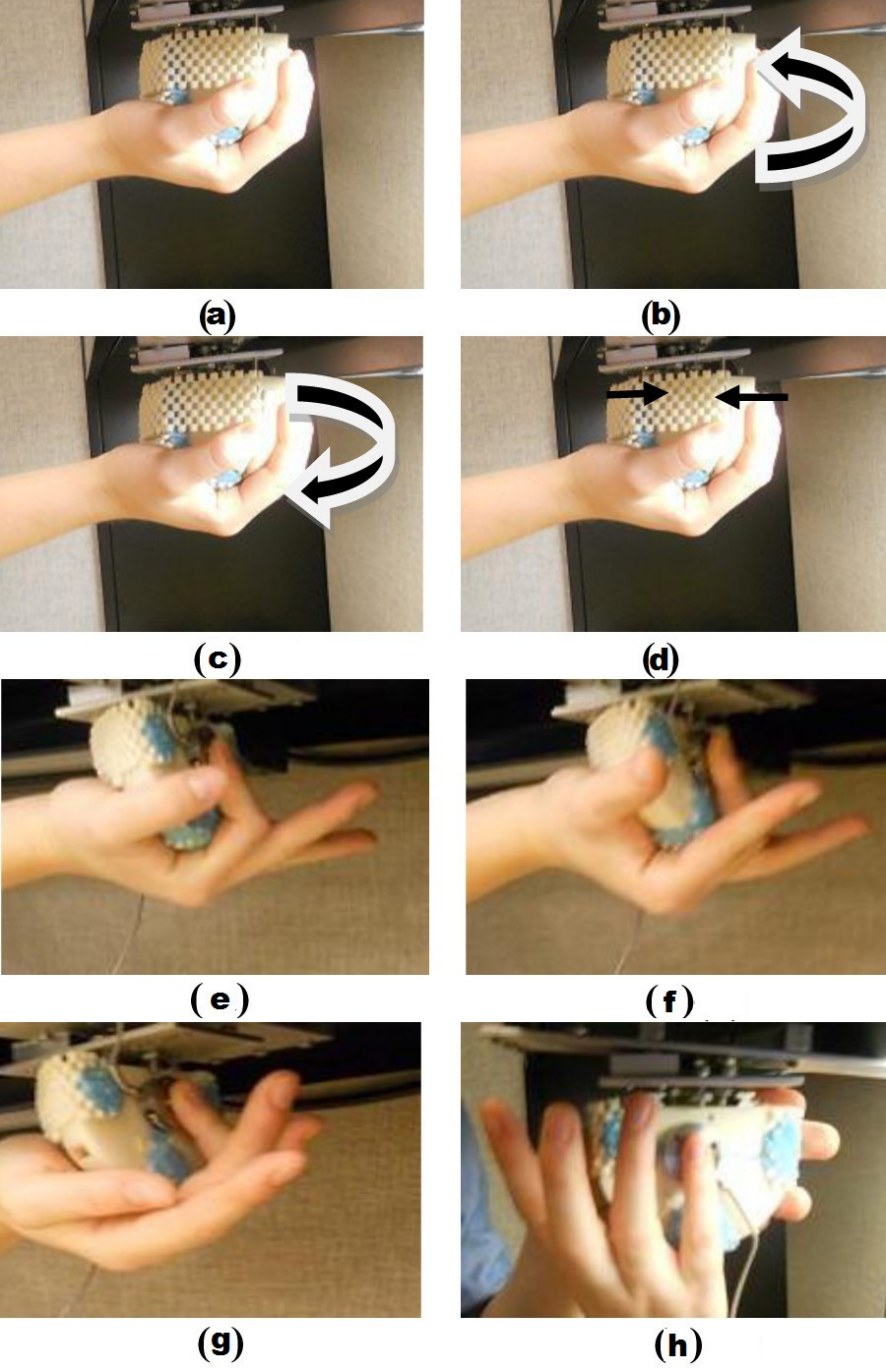
**Hand gestures and motions chosen for classification in the supination position of the arm**. (a) rest, (b) radial deviation, (c) ulnar deviation, (d) grasp, (e) finger pinching: index finger, (f) finger pinching: middle finger, (g) finger pinching: ring finger, (h) finger pinching: little finger.

**Figure 5:**
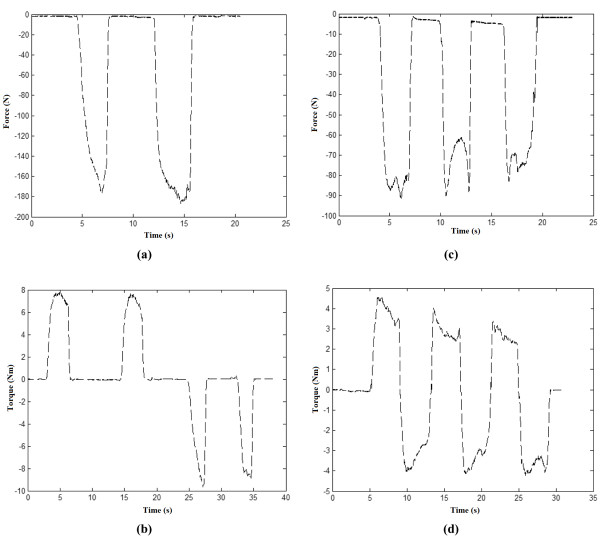
**Forces and torques representing predefined protocols A, B, C and D**. (a) Protocol A, (b) Protocol B, (c) Protocol C and (d) Protocol D.

**Figure 6:**
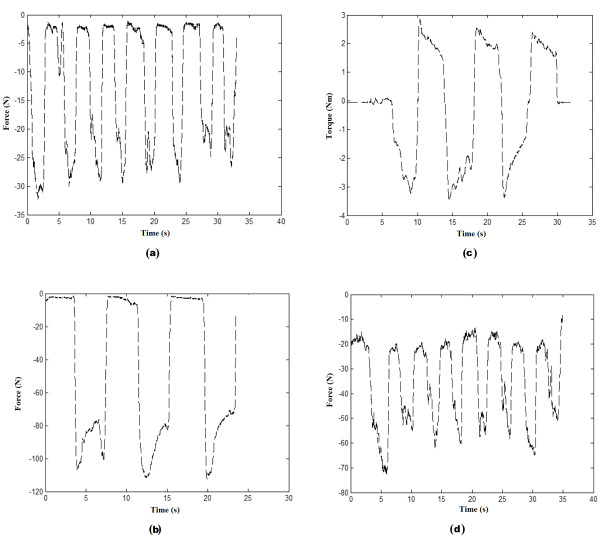
**Forces and torque representing predefined protocols E, FC, FD and FE**. (a) Protocol E, (b) Protocol FC, (c) Protocol FD and (d) Protocol FE.

In Protocols FC, FD and FE (see Figures 4a-h), each volunteer started at rest position and repeated protocols C, D and E but with their arm in supinated position. Figure 5 presents the output recorded by the force and torque sensors for one of the volunteers following protocols A, B, C and D. Figure 6 presents a sample output of the force and torque sensors related to protocols E, FC, FD and FE.

Protocols A and B (see Table 2) were followed to record the maximum torque produced by the user. Protocols C, D, E, FC, FD, and FE were instead used to generate data for the formation of the different hand gesture classes summarized in Table 3. Specifically, protocols C, D and E enabled extracting data for classification purpose in the pronation position of the arm (classes 2-8 in Table 3) whereas protocols FC, FD and FE were used to extract data for classification in the supination position of the arm (classes 9-15 in Table 3).

**Table 3:** Class Definition

Class Number	Class definition
1	Rest

2	Pronation arm position: grasp

3	Pronation arm position: radial deviation

4	Pronation arm position: ulnar deviation

5	Pronation arm position: finger pinching - index finger

6	Pronation arm position: finger pinching - middle finger

7	Pronation arm position: finger pinching - ring finger

8	Pronation arm position: finger pinching - little finger

9	Supination arm position: grasp

10	Supination arm position: radial deviation

11	Supination arm position: ulnar deviation

12	Supination arm position: finger pinching - index finger

13	Supination arm position: finger pinching - middle finger

14	Supination arm position: finger pinching - ring finger

15	Supination arm position: finger pinching - little finger

### Feature extraction and classification

**Figure 7:**
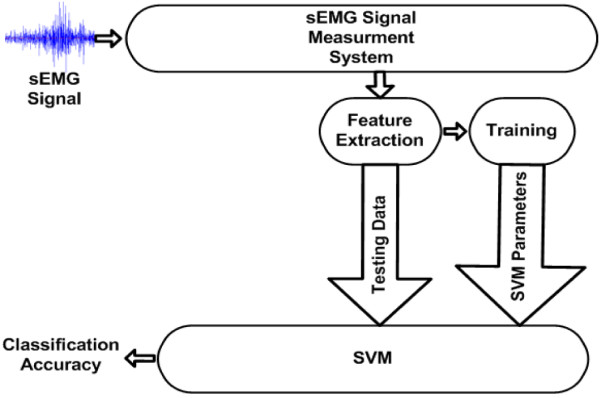
**The proposed sEMG signal classification scheme**.

The proposed sEMG signal classification scheme is presented in Figure 7. As shown in this figure, signals recorded from the Noraxon measurement system were processed in MATLAB R2009a for feature extraction in order to reduce the dimensionality of the raw sEMG input.

Pattern recognition accuracy is influenced by the selection of extracted features and features cannot be extracted from the individual samples as the structural detail of the signal is lost [37]. In fact, the features need to be calculated by segmenting the raw sEMG signal and calculating a set of features from each segment. For this reason, the recorded data was segmented into 250 ms intervals corresponding to 256 samples in each segment and features were extracted from each segment. Then, for the next feature extraction, the segment window was incremented by 125 ms including 128 samples.

Waveform length, time windowed RMS and AR models were used to extract six features for each of the four sEMG channels. Specifically, waveform length and RMS provided one feature each, whereas AR models provided four features in total as explained in the following paragraphs.

The waveform length, which measures the waveform complexity in each segment, was computed as:

(1) $$y=\overset{N}{\underset{r-1}{\sum }}\left|\Delta t_{r}\right|=\overset{N}{\underset{r-1}{\sum }}\left|t_{r}-t_{r-1}\right|$$

where *t_r _*is the amplitude of the *r*^th ^sample and N is the number of samples.

The time windowed RMS value of the raw sEMG signal was used in order to provide information regarding the amplitude of the signal. This feature is mathematically presented as:

(2) $$m_{rms}=\sqrt{\frac{m_{1}^{2}+m_{2}^{2}+\ldots +m_{n}^{2}}{n}}$$

where *m_i _*is the amplitude of the *i*^th ^sample in the time domain, and n is the number of samples. In our case n was equal to 256.

The last feature used in this study was based on AR models. AR models can be defined as a linear combination of previous samples and noise. The mathematical representation of current value is given by (3):

(3) $$t_{n}=\overset{p}{\underset{i=1}{\sum }}q_{i}^{P}t_{n-i}+w_{n}$$

where *w *is the additive noise and {*q *for *i *= 1, ..., *p *} are AR model coefficients. Four AR model coefficients were selected as adequate for modelling EMG signals as discussed in [38].

Six seconds of data per person per protocol was extracted. In order to train and test the pattern recognition model, the gathered data was divided into training and testing sets (see Figure 7) [39]. The testing set was limited to 3807 data segments, namely 90% of the gathered data, as the use of a higher number of segments did not significantly improve the classification accuracy. The remaining 10% of the gathered data, corresponding to 423 data segments, was used as testing set.

SVM [40] was chosen as classifier in this study. SVM was selected among all the other possible pattern recognition tools, as it is a well-known robust classifier, which has extensively and successfully been used to process bio-information signals [41-43]. In addition, SVM works well in high dimensional spaces and has shown good classification results in many practical applications [44-49].

In its general formulation, the SVM [40] requires solving the following optimization problem:

(4) $$\begin{array}{cc} \text{min} & \frac{1}{2}{∥w∥}^{2}+c\overset{N}{\underset{n=1}{\sum }}\xi_{n}\\ \text{subject to} & \begin{array}{c} {\text{a}}_{\text{n}}z\left(x_{n}\right)\geq 1-\xi_{n},\;n=1,...,N\\ \xi_{n}\geq 0 \end{array} \end{array}$$

where *w *is the vector representing adaptive model parameters, *c*>*0 *is the penalty factor, *N *is the total number of data points, *a_n _*is the label associated with a data point, *ξ_n _*is the slack variable, *z *is the learned model, *x_n _*is the vector representing a data point, and *n *is the index associated to a data point.

In this study, the LibSVM tool [50] was used in MATLAB R2009a environment. LibSVM has an implementation for multi class SVM using one-versus-one strategy, whose details are presented in [51]. The LibSVM supports well-known kernels such as the radial basis function (RBF), polynomial, sigmoid and Gaussian kernels.

Following guidelines presented in [52], the RBF was selected as it nonlinearly maps the samples and has limited numbers of hyper parameters thus reducing the complexity of model selection. The mathematical representation of the RBF kernel is:

(5) $$k\left(x_{i},x_{j}\right)=\text{exp}\left(-\gamma {∥x_{i}-x_{j}∥}^{2}\right),\;\gamma >0$$

**Figure 8:**
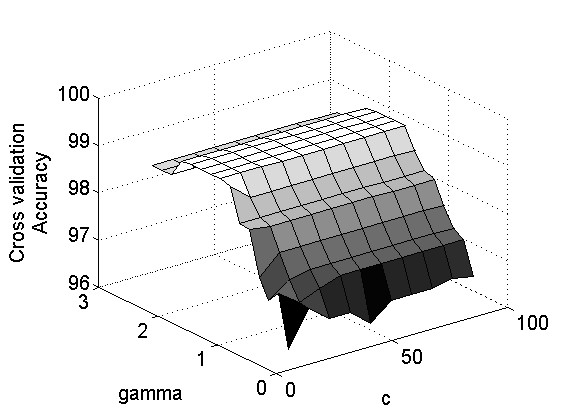
**Cross validation accuracy based on c and γ parameters**.

Eight fold cross validation along with grid search was used to select the pattern recognition optimal parameters *c *and *γ*. Figure 8 shows an illustrative example of results obtained for a single participant. It can be seen that the cross validation accuracy does occur in the interval (0,100) for *c *and (0,3) for *γ*. This interval was selected for the identification of the optimal parameters for all participants.

## Results and discussion

The optimal values for the parameters *c *and *γ *were selected according to the highest value of the cross validation accuracy for each individual volunteer. Table 4 presents the selected *c *and *γ *parameters for each of the twelve seniors (denoted with capital letters A-Q in Table 4) who participated in this study. Each pair of *c *and *γ *parameters was used to build a model for classifying the hand gestures of the participant. Results of the classification accuracies for the 12 seniors are presented in Table 5. An average accuracy of 90.62% was observed.

**Table 4:** The senior cross validation accuracy and model parameters c and γ

Senior	c, γ	Cross validation accuracy (%)
A	10, 1.2	99.17

B	10,1.5	97.92

C	10,0.6	90.42

D	10,0.8	91.67

I	45, 0.4	90.42

K	10,0.6	96.67

L	10,0.4	88.75

M	10,0.5	99.17

N	10,0.6	98.33

O	10,0.4	99.58

P	25, 0.2	95.42

Q	10,2.4	97.92

**Table 5:** The senior pattern recognition accuracy

Senior	Accuracy percentage (%)	Maximum Force (N)	Maximum torque (Nm)
A	91.67	1.79	4.13

B	91.67	2.53	7.03

C	91.67	1.90	5.55

D	87.50	8.84	10.05

I	91.67	1.05	2.14

K	91.67	3.58	7.45

L	83.33	2.12	6.91

M	91.67	3.00	6.45

N	87.50	0.97	1.92

O	91.67	7.01	8.75

P	91.67	1.67	3.58

Q	95.83	2.84	7.10

**Figure 9:**
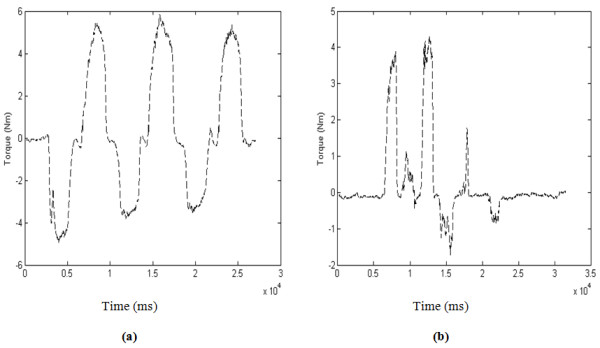
**The output recorded by the torque sensor for seniors**. (a) Senior Q following the protocol FD correctly and (b) Senior L following the protocol FD incorrectly.

The accuracy reached over 95% in the case of the senior Q and less than 85% in the case of the senior L (see Table 5). The senior Q controlled the hand functions well, which resulted in an accurate separation between torque patterns. As an illustrative example, the torque output recorded for the senior Q is shown in Figure 9-a. It is clear from this figure that the senior Q was executing the protocol FD (three repetitions of alternating radial and ulnar deviation). On the other hand, the senior L controlled hand functions poorly, which resulted in small separation between torque patterns. The torque output recorded for the senior L is shown in Figure 9-b; it is clear that this senior was not able to correctly follow protocol FD. It should be noted that, although the classification accuracy was smaller for the senior L (see Tables 5), it was still acceptable (above 83%).

**Figure 10:**
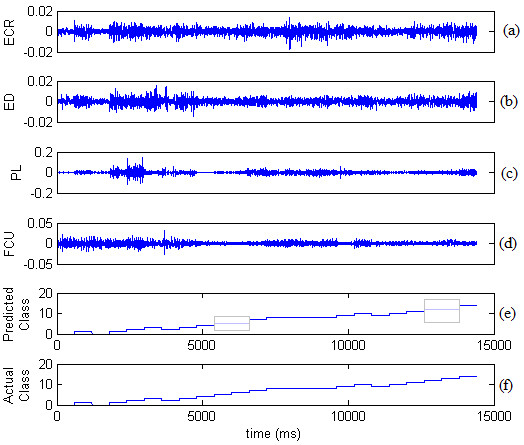
**System performance**. (a) ECR muscle activation, (b) ED muscle activation, (c) PL muscle activation, (d) FCU muscle activation, (e) Predicted class by the system, (f) Actual class.

The system was therefore able to accurately classify the action of the seniors' hand with minimum misclassification, which occurred mainly for finger pinching. Figure 10 shows, for example, sEMG signals extracted from ECR, ED, PL and FCU muscles of senior A (Figures 10a-d), the "predicted classes" identified by our classification system (Figure 10-e) and the "actual classes" corresponding to the different protocols (Figure 10-f). It can be seen that misclassification occurred for consecutive classes related to the finger pinching (see highlighted boxes in Figure 10-e). Specifically, class 7 (ring finger pinching in pronation position) was confused with class 6 (middle finger pinching in pronation position) and class 14 (ring finger pinching in supination position) was confused with class 13 (middle finger pinching in supination position) (see Table 3). It should be noted that this misclassification, which probably resulted by a co-contraction of the forearm muscles, is believed to be acceptable for future potential devices assisting finger movements, as generally middle, ring and little fingers have synergistic patterns during functional grasping [53].

Table 5 also reports the maximum force and the maximum torque each senior was able to exert. The average maximum force was 3.11N and the average maximum torque was 5.92 Nm. No clear relationship was identified between classification accuracy and maximum force or maximum torque exerted by the volunteers. For example, volunteers D and N had equal classification accuracy but their maximum force and torque were respectively the highest and the smallest of the entire group of seniors.

**Figure 11:**
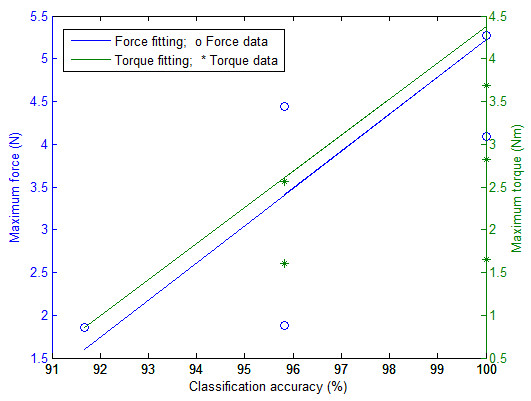
**The relationship between the maximum force/torque and the classification accuracy**.

Table 6 and Table 7 respectively present the selected *c *and *γ *parameters and the corresponding classification accuracies for the group of young volunteers. An average classification accuracy of 97.6% was obtained. Table 7 also reports the maximum force and maximum torque each young volunteer was able to exert. The average maximum force was 4.20N and the average maximum torque was 3.37 Nm. In this case, data suggests a linear relationship between classification accuracy and maximum force and maximum torque, as shown in Figure 11. It should however be noted that the number of young volunteers participating in this study was limited to 7.

**Table 6:** The young volunteer cross validation accuracy and model parameters c and γ

Young volunteer	c, γ	Cross validation accuracy (%)
Y_R	15,0.9	99.17

Y_S	10,0.9	94.58

Y_T	10,1.1	93.33

Y_U	10,0.2	96.67

Y_V	10,0.5	97.50

Y_W	10,0.3	93.75

Y_X	70,0.2	99.58

**Table 7:** The young volunteer pattern recognition accuracy

Young Volunteer	Accuracy percentage (%)	Maximum force (N)	Maximum torque (Nm)
Y_R	91.67	1.86	0.38

Y_S	100.00	5.91	2.65

Y_T	100.00	5.27	5.89

Y_U	95.83	4.44	3.57

Y_V	100.00	4.09	3.82

Y_W	95.83	1.88	2.61

Y_X	100.00	5.90	4.69

A comparison between results obtained for seniors and the young volunteers shows that while maximum force decreased of about 26%, classification accuracy decreased of only 7% with age. Although there are major physical changes occurring in humans while ageing [32], successful sEMG classification is therefore possible in seniors.

## Conclusions

The possibility of associating forearm sEMG patterns to seniors' hand postures was investigated. Results support the hypothesis that successful pattern recognition can be performed to distinguish different hand gestures of seniors in vital activities of daily living.

The identified classes in this study were grasping, radial/ulnar deviation and four different finger pinching in both pronation and supination positions of the seniors' arm. The use of only four sEMG channels demonstrated to be suitable for classifying the fifteen different hand gestures considered in this study. In fact, the implemented pattern recognition strategy was able to identify the different hand gestures with accuracy greater than 90% independently of age and gender. The difference (7%) in classification accuracy observed between the young and older people could be attributed to aging. Misclassification occurred especially in seniors with reduced hand functions. Such a misclassification was however acceptable as it was mainly related to the ring finger, whose use is generally coupled to middle and little fingers during functional grasping.

## Competing interests

The authors declare that they have no competing interests.

## Authors' contributions

MT designed and implemented the feature selection and classification and drafted the manuscript. ZGX collected the data and developed the measurement systems. CM supervised the project, contributed to discussions and analysis and participated in manuscript revisions. All authors read and approved the final manuscript.
